# Anatomical localization of progenitor cells in human breast tissue reveals enrichment of uncommitted cells within immature lobules

**DOI:** 10.1186/s13058-014-0453-3

**Published:** 2014-10-15

**Authors:** Lisa M Arendt, Patricia J Keller, Adam Skibinski, Kevin Goncalves, Stephen P Naber, Rachel J Buchsbaum, Hannah Gilmore, Steven E Come, Charlotte Kuperwasser

**Affiliations:** 1 Developmental, Molecular, and Chemical Biology Department, Sackler School of Graduate Biomedical Sciences, Tufts University School of Medicine, 136 Harrison Ave, Boston, 02111 MA USA; 2 Molecular Oncology Research Institute, Tufts Medical Center, 800 Washington St, Boston, 02111 MA USA; 3 Department of Pathology, Tufts Medical Center, 800 Washington St, Boston, 02111 MA USA; 4 Department of Medicine, Tufts Medical Center, 800 Washington St, Boston, 02111 MA USA; 5 Department of Pathology-Anatomic, UH Case Medical Center, 11100 Euclid Ave, Cleveland, 4410 OH USA; 6 Breast Medical Oncology Program, Beth Israel Deaconess Medical Center, Dana Farber/Harvard Cancer Center, 330 Brookline Ave, Boston, 02215 MA USA; 7 Developmental, Molecular, and Chemical Biology Department, Tufts University School of Medicine, 800 Washington St, Box 5609, Boston, 02111 MA USA

## Abstract

**Introduction:**

Lineage tracing studies in mice have revealed the localization and existence of lineage-restricted mammary epithelial progenitor cells that functionally contribute to expansive growth during puberty and differentiation during pregnancy. However, extensive anatomical differences between mouse and human mammary tissues preclude the direct translation of rodent findings to the human breast. Therefore, here we characterize the mammary progenitor cell hierarchy and identify the anatomic location of progenitor cells within human breast tissues.

**Methods:**

Mammary epithelial cells (MECs) were isolated from disease-free reduction mammoplasty tissues and assayed for stem/progenitor activity *in vitro* and *in vivo*. MECs were sorted and evaluated for growth on collagen and expression of lineages markers. Breast lobules were microdissected and individually characterized based on lineage markers and steroid receptor expression to identify the anatomic location of progenitor cells. Spanning-tree progression analysis of density-normalized events (SPADE) was used to identify the cellular hierarchy of MECs within lobules from high-dimensional cytometry data.

**Results:**

Integrating multiple assays for progenitor activity, we identified the presence of luminal alveolar and basal ductal progenitors. Further, we show that Type I lobules of the human breast were the least mature, demonstrating an unrestricted pattern of expression of luminal and basal lineage markers. Consistent with this, SPADE analysis revealed that immature lobules were enriched for basal progenitor cells, while mature lobules consisted of increased hierarchal complexity of cells within the luminal lineages.

**Conclusions:**

These results reveal underlying differences in the human breast epithelial hierarchy and suggest that with increasing glandular maturity, the epithelial hierarchy also becomes more complex.

**Electronic supplementary material:**

The online version of this article (doi:10.1186/s13058-014-0453-3) contains supplementary material, which is available to authorized users.

## Introduction

The breast undergoes dynamic changes over the lifetime of a woman, from initial development at puberty, to proliferation and apoptosis during the menstrual cycle in response to hormonal fluctuations, and culminating with full lobuloalveolar development for lactation. Studies from murine mammary glands have demonstrated the presence of an epithelial hierarchy that serves to prevent exhaustion of progenitors necessary for tissue homeostasis and glandular regeneration. These extensive studies have identified and localized lineage-restricted progenitor cells that can be assayed using both *in vitro* and *in vivo* techniques (for review [1]-[4]).

There are numerous differences between the human breast and the mouse mammary gland that preclude the direct translation of rodent studies to human breast development. The human breast is composed of 11 to 48 central ducts that radiate outward from the nipple [5], where circulating hormones and localized growth factors likely coordinate the growth of the terminal ductal lobular units (TDLU) that emanate from primary ducts. Each lobe is organized as heterogeneous lobular structures, each one representing a sequential developmental stage [6]-[8]. In contrast, mouse mammary glands are composed of a simple ductal tree that lack TDLU, and only exhibit strain-specific rudimentary alveolar budding in the absence of pregnancy. Thus, the anatomical and physiological equivalency of lobules and TDLU is unclear in the mouse.

The mammary gland is composed of a bi-layered epithelium; basal/myoepithelial (ME) cells express cytokeratin (CK) 14 surrounding a luminal layer that stains positively for CK8/18. In the mouse, CK expression is specific for each epithelial layer, which has enabled the use of these markers for lineage tracing studies to establish the presence of lineage-restricted progenitor cells within each layer of the mammary epithelium [9]. Unlike the mouse, little is known about the identity and dynamics of progenitor cells in the human breast, and details about their activity and the mechanisms that regulate their numbers and differentiation remain poorly understood. Interestingly, contiguous regions of human breast lobules contain cells showing identical X-chromosome inactivation patterns suggesting that they were likely derived from a common uncommitted stem cell [10].

The limited understanding of human breast development and stem cell biology has largely been due to the lack of appropriate model systems and assays to detect, analyze, and characterize stem cell properties. In recent years, we and others have developed and optimized various *in vivo* and *in vitro* tools to study the biology and mechanisms governing human breast development [1],[11]-[16]. Using these approaches we sought to dissect the epithelial hierarchy of the human breast and identify the anatomic locations of progenitor cells within the breast. In doing so, we reveal that human breast tissues contain two types of phenotypically distinguishable progenitor cells localized to the luminal and basal lineages, respectively, which contribute to different anatomical structures. Further, we show that immature lobules within the breast harbor distinct types of progenitor cells.

## Materials and methods

### Animal studies

All animal procedures in this study were approved by the Tufts University Institutional Animal Care and Use Committee (IACUC), and all animal procedures were conducted in accordance with this approved protocol. Colonies of NOD/SCID mice were maintained in house. Mice were given food and water *ad libitum*.

### Primary tissue isolation and culture

All human breast tissues were obtained in compliance with the laws and institutional guidelines, as approved by the Institutional Review Boards (IRB) from Tufts Medical Center and Beth Israel Deaconess Medical Center. For these studies, we utilized de-identified, non-cancerous breast tissues from patients undergoing elective reduction mammoplasty surgery under the Category 4 exemption of the IRB Review. As de-identified patient samples were utilized for this study, patient consent was not required. The tissue was sampled to make whole mounts and paraffin blocks, and snap frozen for molecular analyses, and the remainder was enzymatically digested to epithelial organoids as previously described [12],[17]. The epithelial organoids were aliquoted in 1:1 DMEM/Hams-F12 media (Invitrogen, Grand Island, NY USA) supplemented with 5% calf serum, 10 ng/mL insulin, 10 μg/mL epidermal growth factor (EGF), 10 μg/mL hydrocortisone, and 10% dimethyl sulfoxide (DMSO) and stored in liquid nitrogen for later use Insulin, hydrocortisone, EGF, and DMSO were all obtained from Sigma-Aldrich Corporation (St. Louis, MO, USA). To humanize mice, mammary epithelium was removed from the fourth mammary glands of 3-week-old NOD/SCID females, and RMF-EG cells were injected into the fat pad as described [12]. Two weeks post-humanization, epithelial organoids were dissociated to single cells (100,000 cells), co-mixed with primary breast stromal cells (2.5 × 10^5^ cells per gland) in a 1:1 mixture of collagen and Matrigel (BD Biosciences, San Jose, CA, USA) and injected into humanized fat pads. RMF-EG cells and primary breast stromal cells were grown in high glucose DMEM (Invitrogen) supplemented with 10% calf serum and 1% penicillin/streptomycin at 37°C and 5% carbon dioxide.

### Progenitor cell assays

For colony formation, 40,000 human mammary epithelial cells (MEC) were plated on non-adherent plates or adherent plates in 2 mL of epithelial basal media supplemented with bovine pituitary extract (52 μg/mL), hydrocortisone (0.5 μg/mL), human EGF (10 ng/mL), and insulin (5 μg/mL; Lonza, Allendale, NJ, USA) for 7 days. Mammospheres or floating colonies were plated in triplicate and quantified using a Multisizer3 Coulter Counter (Beckman Coulter, Danvers, MA, USA). Colonies growing on adherent plates were fixed with methanol and stored at -20°C until stained for cytokeratins 8 and 14. Mammospheres and floating colonies were cytospun onto glass slides, methanol fixed, and stored at -20°C until used for analyses. For growth on collagen, 1 mg/mL rat tail collagen (pH = 7; BD Biosciences) polymerized for 30 minutes at 37°C on four- or eight-well chamber slides (Corning, Corning, NY, USA). MECs (n = 5,000) were plated in 1 mL of complete epithelial basal media supplemented with 2% Matrigel (BD Biosciences) in duplicate. Colony growth was observed using Nikon Eclipse Ti and quantified based on morphology. For mammosphere and floating colony growth on collagen, MECs were grown as mammospheres and floating colonies for 7 days in triplicate. One replicate was quantified, and two replicates were plated on collagen. All conditions were plated on collagen in duplicate.

### Mammary whole mounts

Tissue isolated from elective reduction mammoplasty surgery from eight patients was used to make whole mounts. Each tissue was sampled in 5 to 10 different areas to make whole mounts, which were formalin-fixed overnight and stained with carmine. The tissue was sliced into 2- to 3-mm sections, dehydrated in graded alcohols, and incubated in xylenes to remove fat. The mammary sections were imaged using a Nikon Eclipse80i microscope, and lobules were identified as described [6]. Briefly, the number of ductules/lobule were counted and categorized as Type I (average (avg) 11 ductules/lobule), Type II (avg 47 ductules/lobule), and Type III (avg 81 ductules/lobule). Lobules of each type were quantified from multiple sections of the reduction mammoplasty tissue, bluntly dissected with a razor, paraffin-embedded, and sectioned at 5 μm for immunohistochemical and immunofluorescent staining.

### Immunofluorescence and immunohistochemistry

Human-in-mouse normal outgrowth tissue sections were stained with an antibody for α-smooth muscle actin (1:1000; Vector Laboratories, Burlingame, CA, USA). Sorted breast lobules and sections from human-in-mouse normal outgrowths were stained with antibodies for cytokeratin (CK) 8/18 (1:250, Vector Laboratories) and CK 14 (1:250, Thermo Scientific, Tewksbury, MA, USA). Breast lobules were additionally stained with p63 (1:500; Santa Cruz Biotechnologies, Dallas, TX, USA), estrogen receptor alpha (1:500, F10 clone, Santa Cruz Biotechnologies), progesterone receptor (1:250; Cell Signaling Technologies, Danvers, MA, USA), and EpCAM (1:500; Stem Cell Technologies, Vancouver, BC, Canada).

For immunohistochemistry, tissue sections were incubated with 2% hydrogen peroxide to quench endogenous peroxidase, antigen retrieved in 0.1 M citrate (pH = 6.0), and blocked with 1% BSA and 1.5% goat serum in PBS for 1 hour. Tissue sections were incubated with primary antibodies in 1% BSA in PBS overnight at 4°C, followed by secondary antibodies for 30 minutes at room temperature (1:250 biotinylated anti-mouse or anti-rabbit; Vector Laboratories). Staining was detected using Vectastain ABC kit followed by ImmPact DAB kit (Vector Laboratories), and sections were counterstained using hematoxylin. For adherent colonies on plastic plates, colonies were fixed in methanol at −20°C for 10 minutes, and cells were permeabilized with 0.1% triton in PBS for 10 minutes. Cells were blocked with 1% bovine serum albumin (BSA) and 1.5% goat serum in PBS for 1 hour, incubated with anti-rabbit CK 14 at 4°C overnight, and stain was detected as described above. Following 3,3-diaminobenzidine (DAB) stain, colonies were blocked with the avidin/biotin blocking kit (Vector Laboratories) per the manufacturers’ instructions, with 1% BSA in PBS for 1 hour, and with anti-mouse CK 8/18 overnight. Specific staining was detected as described above, except the substrate used for detection was the VIP kit (Vector Laboratories). Colonies were air-dried and quantified by morphology and cytokeratin expression as described [16].

For immunofluorescence, tissue sections were antigen-retrieved in 0.1 M citrate (pH = 6.0) and blocked with 1% BSA and 1.5% goat serum in PBS for 1 hour. Tissue sections were incubated with primary antibodies in 1% BSA in PBS overnight at 4°C, followed by secondary antibodies, for 30 minutes at room temperature (1:250; Alexa Fluor 488 or 546, Invitrogen). Nuclei were counterstained with 4′,6-diamidino-2-phenylindole (DAPI) in the mounting media using Vectashield (Vector Laboratories). For double-labeling, tissue sections or mammospheres and floating colonies were incubated with rabbit primary antibodies and secondary antibodies followed by mouse primary and secondary antibodies. Images were captured with Nikon Eclipse 80i and merged using ImageJ software.

### Quantification of mammary whole-mount staining

Five lobules of each type were evaluated from each patient sample. The percentage of positive cells for each marker was quantified in each high-power image and categorized into one of three percentage categories. EpCAM staining was evaluated using Allred scoring based on staining intensity and number of cells staining positively. Staining differences among lobule types was determined by chi-squared analyses.

### Flow cytometry

Unsorted cells from organoid preparations were dissociated to a single-cell suspension as described above and filtered through a 20 μm nylon mesh (EMD, Millipore, Billerica, MA, USA). Endothelial, lymphocytic, monocytic, and fibroblastic lineages were depleted with antibodies to CD31, CD34, and CD45 (all from Thermo Scientific), fibroblast-specific protein IB10 (Sigma), and a mixture of pan-mouse IgG and IgM Dynabeads (Dynal; Invitrogen) according to the manufacturers’ instructions and as described previously [11],[18]. Lineage-depleted single-cell suspensions or cells from immunomagnetic bead-sorted populations were resuspended at 1 × 10^6^ cells per mL in PBS containing 1% calf serum (fluorescence-activated cell sorting (FACS) buffer) and bound with fluorescently conjugated antibodies to human EpCAM (allophycocyanin), CD24 (FITC; BD Biosciences), CD49f (PerCP-Cy5.5; Biolegend, San Diego, CA, USA), and CD10 (phycoerythrin; Dako, Carpenteria, CA, USA) for 20 minutes at 4°C. Antibody-bound cells were washed and resuspended at 1 × 10^6^ cells per mL in FACS buffer and run on a FACSCalibur flow cytometer (BD Biosciences). Flow cytometry data were analyzed with the FlowJo software package (TreeStar, Ashland, OR, USA). Cellular debris and dead cells removed from analysis through manual gating and use of propidium iodide. Gates were set on the remaining cells based on isotype controls for each antibody.

### Spanning-tree progression analysis of density-normalized events (SPADE)

SPADE was used to generate tree diagrams from flow cytometry data as previously described [19] using the SPADE 2.0 MATLAB implementation. Enzymatically dissociated, lineage-depleted breast-reduction tissue was prepared and analyzed by flow cytometry. Ungated FACS data generated from eight patient samples was used to construct the SPADE tree using three parameters (CD49f, CD24, and EpCAM). Downsampling was manually set at 10,000 cells per sample and the number of desired clusters set to 50. The SPADE tree generated was evaluated and sectioned based on characterized relationships between markers: mature luminal (EpCAM^hi^CD49f^-^), luminal progenitor cells (LPC) (EpCAM^hi^CD49f^+^), mature basal (MB) (EpCAM^lo^CD49f^+^), basal progenitor cells (BPC) (EpCAM^-^CD49f^+^), and mammary lineage negative (MLN) (EpCAM^-^CD49f^+^). To visualize differences between tissue samples predominantly consisting of immature (Type I/II) and mature (Type III) lobules, data from four patients enriched for Type I/II lobules and 4 patients enriched for Type III lobules were concatenated in FlowJo, upsampled to the previously generated SPADE tree, and pseudocolored to indicate differences in the frequency of cells falling into each cluster.

### Bead sorting

MEC were plated briefly in serum (1 to 2 h) to deplete mammary fibroblasts from the organoid fraction. The organoids remaining in suspension were dissociated by trypsinization and filtered with a 40 μm filter (BD Biosciences) to remove residual clustered cells. Single-cell suspensions of breast epithelial cells were sorted with CELLection pan-mouse IgG magnetic beads (Dynal; Invitrogen) conjugated to an anti-CD10 antibody (clone SS2/36; Santa Cruz Biotechnology) according to the manufacturer’s instructions. CD10^+^ cells were released from the beads by DNase treatment with occasional agitation at 37°C for 10 minutes. Cells that did not bind to the CD10 beads were further sorted with magnetic beads conjugated to an anti-EpCAM antibody (clone VU-ID9; Abcam, Cambridge, MA, USA and AbD Serotec, Raleigh, NC, USA). Positive cells were again released by DNase treatment. EpCAM^+^ bead-sorted cells were further sorted by binding of CD49f antibody (clone 450-30A; AbD Serotec) followed by binding of pan-mouse IgG CELLection beads. Beads were released from positively sorted cells as described above. Viable cells (verified by trypan blue exclusion) from unsorted, basal progenitor cells (BPC, EpCAM^-^CD10^+^), mature basal (MB; EpCAM^+/lo^CD10^+^), luminal progenitor cells (LPC, EpCAM^+^CD49f^+^) and mature luminal (ML) (EpCAM^+^CD49f^-^) cells were used for collagen assays.

### Statistics

Differences between two groups were detected with Student’s *t*-test, and differences among multiple groups were detected using repeated measures analysis of variance (ANOVA) with multiple comparisons post hoc test. Statistical analyses were performed using Graph Pad Prism software (La Jolla, CA, USA).

## Results

### Heterogeneity in human breast epithelial cell lineages

**Figure 1:**
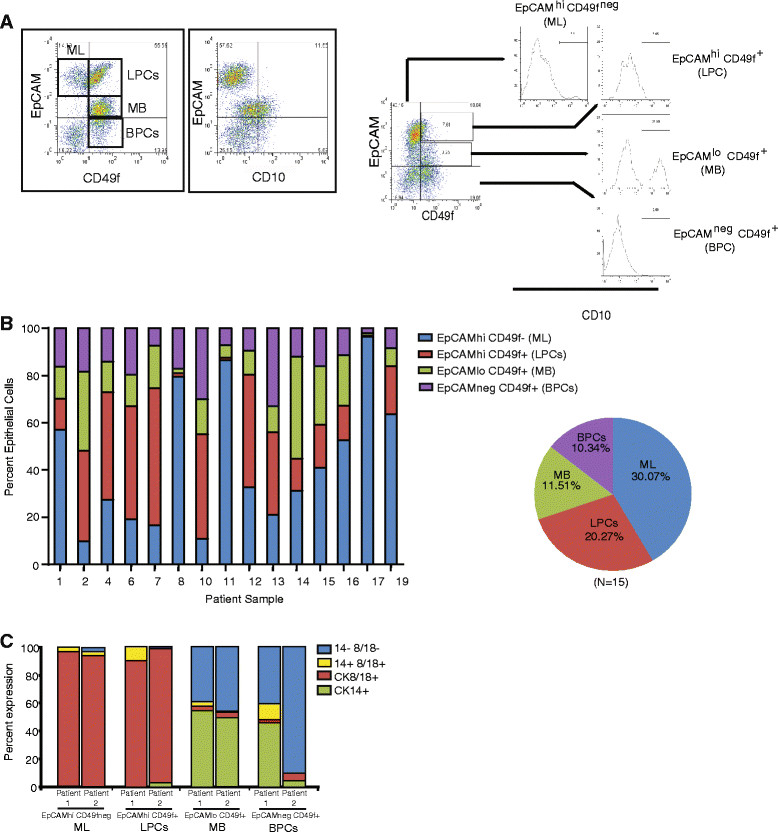
**Cell surface markers define cell populations that are variable among patient-derived epithelial cells. (A)** Representative flow cytometry plots demonstrating defined epithelial cell populations using the cell surface markers EpCAM, CD10 and CD49f. ML = mature luminal, LPC = luminal progenitor cells, MB = mature basal, and BPC = basal progenitor cells. Epithelial cells were isolated from breast tissue from patients undergoing elective reduction mammoplasty surgery. **(B)** Percentage of epithelial cells in each cell population for 15 patient samples. **(C)** Epithelial cells were sorted from primary breast tissue using cell surface markers EpCAM and CD49f and stained for cytokeratin 8 (CK8) and CK14. ML and LPC populations were enriched for CK8^+^ epithelial cells, and MB and BPC were enriched for CK14^+^ cells.

In humans, the surface antigen markers EpCAM and CD49f have been used to define MEC populations within the luminal and basal lineages [11],[20]-[22]. Specifically, cells with an EpCAM^+hi^/CD49f^neg^, EpCAM^+hi^/CD49f^+^, EpCAM^+lo^/CD49f^+^, and EpCAM^neg^/CD49f^+^ immunophenotype are enriched for ML, LPC, MB, and BPC, respectively (Figure 1A). Similarly, the cell surface marker CD10/CALLA is expressed by basal/myoepithelial (MB) cell populations (Figure 1A); [11],[23]-[26]. Using these markers, we performed flow cytometry on lineage depleted epithelial cells isolated from tissue generated from elective reduction mammoplasty surgery (n = 15 patient samples, characterized in Additional file 1: Table S1), and we quantified the ML, LPC, MB, and BPC populations. Significant heterogeneity in the percentage of ML, LPC, MB, and BPC populations was observed in the normal breast tissue across all patient samples (Figure 1B, Additional file 1: Table S1 and Table S2) and was not correlated with age or parity.

To characterize the ML, LPC, MB, and BPC populations, MEC were sorted based on EpCAM and CD49f expression and assessed for luminal and basal lineage markers (CK8 and CK14, respectively). Although all EpCAM^hi^ cells expressed abundant CK8, MLs and LPCs also contained sub-populations that co-expressed both CK8 and CK14 markers (Figure 1C). Similarly, while the majority of sorted MB and BPC lacked CK8 expression, there was a sub-population of EpCAM^lo/neg^/CD49f^+^ cells that co-expressed both CK8 and CK14 markers (Figure 1C). These results suggest that CK8 expression is not exclusively restricted to the luminal lineages in human MEC.

### Quantification of progenitor activity from human breast tissues

**Figure 2:**
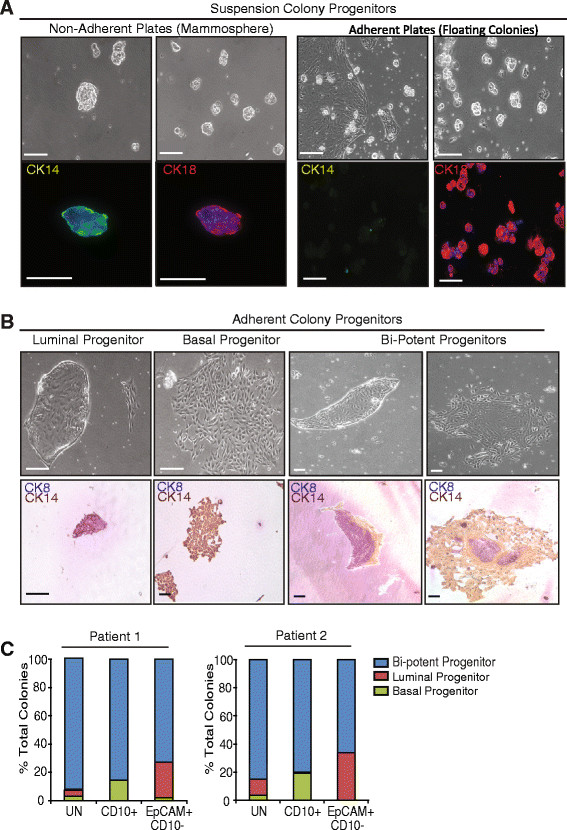
**Epithelial cell growth in suspension enriched for progenitors in different lineages. (A)** Colonies from epithelial cells that grew in suspension in non-adherent culture (mammospheres) expressed both cytokeratin 8 (CK8^+^; luminal) and CK14 (basal). Colonies that grew in suspension over adherent plates (floating colonies) were CK8^+^ but were negative for CK14. **(B)** Epithelial cells formed characteristic colonies on adherent plates that were CK8^+^, CK14^+^, or CK8/14^+^ (bi-potent). **(C)** When grown on adherent plates, CD10^+^ cell populations formed bi-potent and basal colonies, while EpCAM^+^ cell populations formed bi-potent and luminal colonies. Epithelial cells were sorted based on cell surface markers EpCAM and CD10, grown in adherent culture, stained for CK8/14, and colonies were quantified. Scale bar = 100 μm.

Although flow cytometry studies have identified epithelial populations that correspond to distinct lineages within the epithelial hierarchy, they do not directly quantify progenitor activities. *In vitro* and *in vivo* assays have been established to quantify progenitor numbers and to assess functional activity of dissociated MECs isolated from reduction mammoplasty tissue. When grown at clonal density under non-adherent culture conditions, MECs formed mammospheres that were enriched for bi-potent CK8/14^+^ (Figure 2A; [27]) cells, suggesting that both luminal and basal lineages of cells were enriched. Mammosphere progenitor frequency was 6.7/1000 cells when quantified in 19 MEC samples (Table 1, Additional file 1: Table S3). In contrast, when grown on adherent plates, luminal MEC preferentially floated in suspension and formed colonies that were enriched for CK8^+^ cells under these culture conditions (Figure 2A; [11]), suggesting that these floating colonies are enriched for luminal progenitor activity. The frequency of luminal progenitor growth was 4.8/1000 cells (n = 18 MEC samples; Table 1, Additional file 1: Table S3).

**Table 1:** **Summary of frequency of progenitor activity**

Uncultured cells			
Progenitor type	Progenitor lineage	mean ± sd	Frequency, number/1000 cells
Adherent progenitors (n = 19)			
Total	Both	0.0098 ± 0.0023	9.8/1000
Bi-potent	Both	0.002 ± 0.0014	2/1000
Luminal progenitors	Luminal	0.0036 ± 0.004	3.6/1000
Basal progenitors	Basal	0.0029 ± 0.004	2.9/1000
Non-adherent progenitors			
Mammospheres (n = 19)	Both	0.0067 ± 0.00092	6.7/1000
Floating colonies (n = 18)	Luminal	0.0048 ± 0.00088	4.8/1000
Structural progenitors (n = 13)			
Total		0.003 ± 0.0006	3/1000
Luminal alveolar progenitor	Luminal	0.002 ± 0.003	2/1000
Basal ductal progenitor	Basal	0.0009 ± 0.0004	0.9/1000

Average age 34.5 ± 12.6 yrs (mean ± SD).

While a population of luminal progenitor cells formed floating colonies, primary MEC also formed adherent colonies enriched for luminal (CK8^+^), basal (CK14^+^), or bipotent cells (CK8/14^+^; Figure 2B, [11],[16]). There was significant patient heterogeneity in the predominant colony type that grew on plastic. Luminal and basal colonies formed the most frequently, 3.6/1000 and 2.9/1000 cells respectively (n = 19 MEC samples; Table 1, Additional file 1: Table S3), while mixed-lineage colonies occurred at a lower frequency (2/1000 cells).

To determine how this colony-forming ability related to the ML, LPC, MB, and BPC populations delineated by flow cytometry, MEC were sorted for EpCAM and CD10 expression and grown on adherent plates to examine lineage markers of the resulting colonies (luminal CK8 and basal CK14). Sorted luminal (EpCAM^+^) cells rarely attached to adherent plates, but when they did, they formed colonies enriched in luminal and bi-potent cells (Figure 2C). While basal cells (CD10^+^) readily formed colonies, they formed colonies enriched for basal as well as bi-potent cells (Figure 2C). These results suggest that CK8/14 bi-potent progenitor cells are present within both luminal and basal epithelial lineages of the human breast.

### Structural progenitor activity is present in luminal and basal lineages

**Figure 3:**
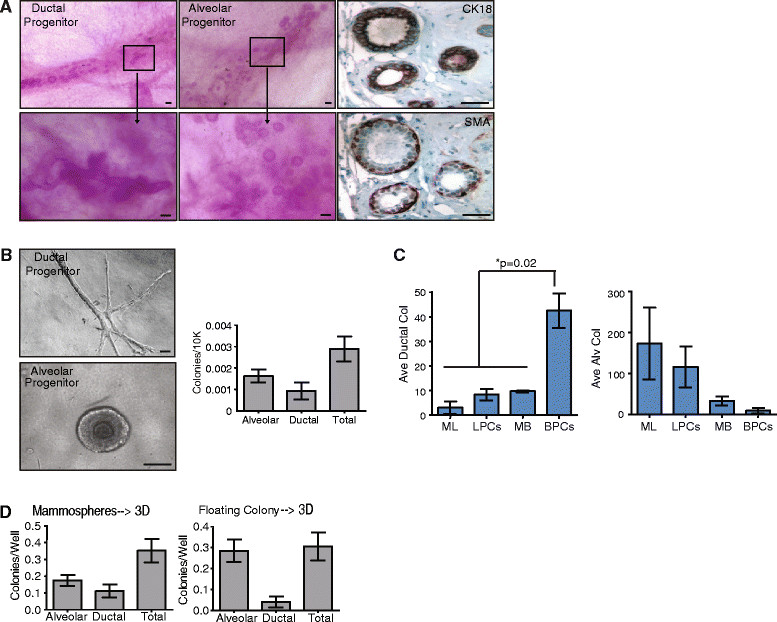
**Alveolar progenitors are enriched in luminal cell populations, while ductal progenitors are enriched in basal cell populations. (A)** *In vivo*, primary epithelial cells isolated from reduction mammoplasty tissue form bilayered ductal or alveolar structures when grown in the humanized fat pads of NOD/SCID mice. These structures expressed luminal cytokeratin 18 (CK18) and basal smooth muscle actin (SMA). **(B)** *In vitro*, primary epithelial cells formed either ductal or alveolar structures when grown on collagen gels (n = 12; mean ± standard error of the mean (SEM)). **(C)** Ductal progenitor activity was enriched in the basal progenitor cell (BPC) population, while alveolar progenitor activity was enriched in mature luminal (ML) or luminal progenitor cell (LPC) populations. Epithelial cells were sorted using cell surface markers CD10 and EpCAM and grown on collagen gels. Differences were detected using analysis of variance (n = 3; mean ± SD). **(D)** Growth in suspension as mammospheres enriched for both alveolar and ductal progenitor activity, while growth in suspension as floating colonies over adherent plates enriched for alveolar progenitor activity. Epithelial cells were grown in suspension as floating colonies and mammospheres for 7 days then plated on collagen gels. Ductal and alveolar progenitor activity was quantified (n = 12; mean ± SEM). Scale bar = 100 μm. MB, mature basal.

In order to examine human MEC growth *in vivo*, we dissociated MEC and implanted them into humanized mammary fat pads of NOD/SCID mice. Dissociated MEC demonstrated the ability to form bi-layered ductal or alveolar structures (Figure 3A), that were positive for CK18 in the luminal layer as well as smooth muscle actin (SMA), which is expressed by myoepithelial cells (Figure 3A). *In vitro*, single MECs grown at clonal density also formed distinct ductal and alveolar colonies (Figure 3B), when grown on a Type I collagen substrate three-dimensionally. The frequency of these structural progenitors was quantified from dissociated MEC from 13 patient samples (Figure 3B, Table 1).

To assess which types of structural progenitors were enriched within sorted MEC populations, ML, LPC, MB, and BPC were sorted based on EpCAM and CD10 expression and were grown at clonal density on collagen. Sorted ML and LPC populations were enriched in alveolar progenitors, while BPC were the major source of ductal progenitors (Figure 3C), consistent with recent findings [11]. These results suggest that both ML and LPC populations are enriched for alveolar progenitor cells, while the BPC population is the major source for ductal progenitor cells.

In order to evaluate the relationship between luminal and basal progenitors and structural progenitors, we grew primary MECs for 7 days in non-adherent cultures (mammospheres) or in suspension above adherent plates (floating colonies) and then quantified and plated the colonies on collagen gels (Figure 3D). Growth as mammospheres significantly enriched both alveolar (85-fold) and ductal progenitor cells (275-fold) compared with growth as single cells on collagen. In contrast, growth as floating colonies only significantly enriched alveolar progenitor cells (140-fold). Together, these results suggest that growth of MEC as mammospheres enriches for both luminal alveolar and basal ductal progenitor activity, while growth as floating colonies preferentially enriches for luminal alveolar progenitor activity.

### *In situ*localization of lineage restricted and unrestricted cells within lobules

**Figure 4:**
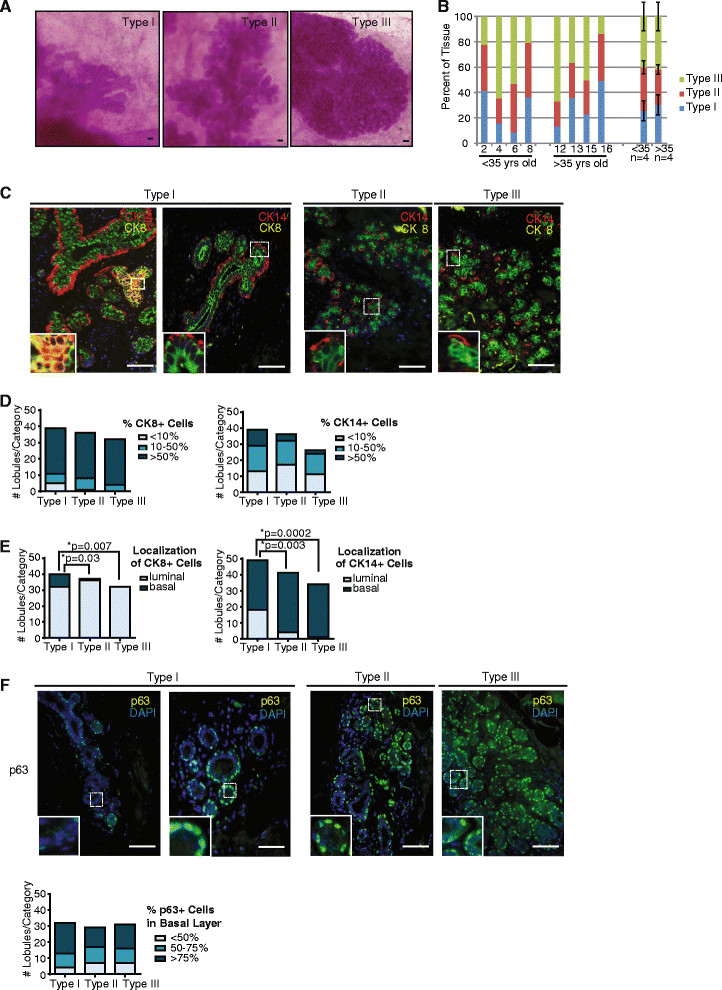
**Type I lobules demonstrated variable cytokeratin 8 (CK8) and CK14 expression. (A, B)** Type I, Type II, and Type III lobules were **(A)** identified and microdissected from reduction mammoplasty tissue, then **(B)** quantified in each tissue sample (n = 8). **(C,** **D)** No significant differences were detected in the percentage of cells expressing CK8 and CK14 among lobule types. Type I-III lobules were stained for CK8 and CK14 and characterized for the percent positive cells in each lobule. The number of individual lobules in each category was quantified for each lobule type (n = 15 lobules/patient). **(E)** Type I lobules demonstrated significantly increased expression of basal CK8 and luminal CK14 compared to other lobule types. Type I-III lobules were characterized for expression of CK8 and CK14 in the luminal and basal layer, and the number of individual lobules in each category was quantified for each lobule type (n = 15 lobules/patient). Statistical differences were detected by chi squared analysis. **(F)** p63 was expressed exclusively in the basal layer in all lobules types examined. Type I-III lobules were characterized for the percentage of cells in the basal layer that expressed p63. The number of individual lobules in each category was quantified for each lobule type (n = 15 lobules/patient). Scale bar = 100 μm.

Although human breast stem/progenitor cells have been proposed to exist within both main ducts and TDLU, it is unclear in which ducts and TDLU these progenitor cells actually reside. Human breast lobules have previously been categorized as Type I, Type II, Type III, and Type IV based on the complexity of the ductule/lobule formation. Type I lobules are the least developed, having the smallest number of ductules/lobules, while Type IV are the most developed and are only present in the breast during pregnancy and lactation [6],[8]. To determine whether each of the lobule types might represent anatomical structures that harbor different types of progenitor cells, we microdissected lobules from human breast reduction mammoplasty samples from women from ages 18 to 50 years and quantified the different types of lobules in each patient sample, according to the previously described system [6]. As expected, there was considerable heterogeneity among patient samples in the number of Type I, Type II and Type III lobules present in any given breast tissue (Figure 4A, B). In contrast to previous reports [8], there was no correlation between the number and types of lobules and age; when averaged across all breast tissue, Type I, Type II and Type III lobules represented about 1/3 of the structures (Figure 4B). Interestingly, patients number 4 and 6 demonstrated increased numbers of Type III lobules (Figure 4B), although both patients were nulliparous (Additional file 1: Table S1). In contrast, patient 13 had fewer Type III lobules following three pregnancies (Figure 4B, Additional file 1: Table S1).

Given that the lobules are thought to undergo successive maturation [6],[8], we examined markers to assess the maturity of each lobule type. Type I lobules demonstrated the most variability with respect to CK8 and CK14 expression; in 11% of Type I lobules <10% of cells stained positive for CK8 expression while this was rarely observed in Type II and Type III lobules. In addition, in 28% of Type I lobules >50% of cells stained positive for CK14 while such high levels of CK14 expression were not found in Type II and Type III lobules (Figure 4C, D). Furthermore, unlike the murine mammary gland [9], CK8 and CK14 expression was not always restricted to the luminal and basal layers of the epithelium, respectively, while p63, a well-characterized marker of basal epithelial cells, exhibited restricted basal cell expression within all of the lobules examined (Figure 4F). In Type I lobules, basal cells expressed CK8 and luminal cells expressed CK14. In fact, nearly 40% of Type I lobules demonstrated luminal CK14 expression, and epithelial cells were identified that co-expressed CK8 and CK14 (Figure 4C, E). In addition, unlike Type II and Type III lobules, Type I lobules exhibited a more heterogeneous distribution of CK expression. While Type II and III lobules were significantly enriched in luminal CK8^+^ cells they contained fewer basal CK14^+^ cells. Moreover, the epithelial cells within Type II and Type III cells showed a predominant linage-restricted expression pattern of luminal and basal markers (Figure 4C, D). These results suggest that Type I lobules are enriched for fewer lineage-restricted progenitor breast epithelial cells and contain more CK14^+^ and CK8^+^ epithelial cells.

### Type I lobules demonstrate reduced expression of luminal markers

**Figure 5:**
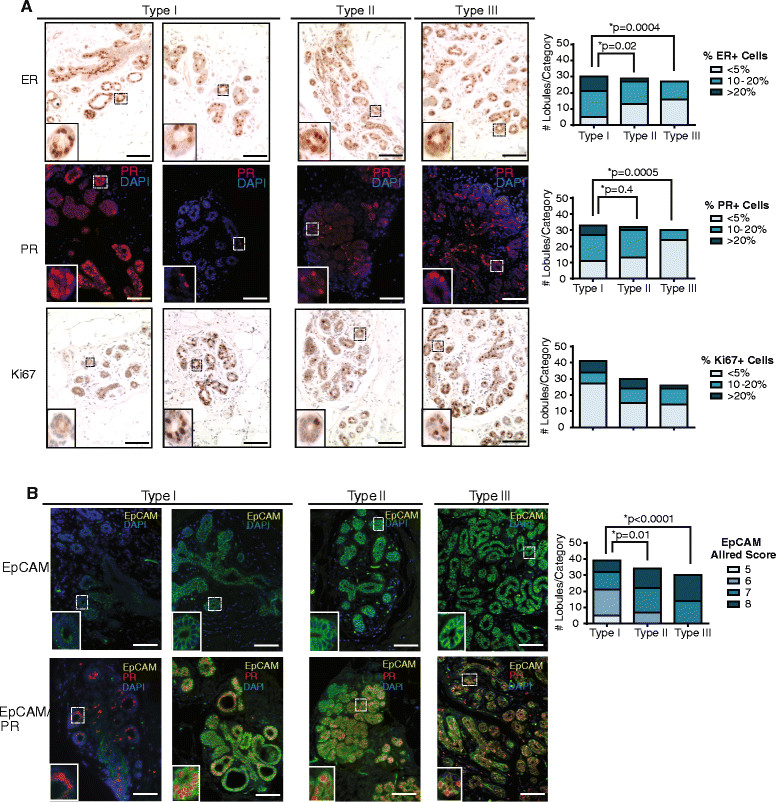
**Type I lobules are enriched for basal progenitor cells. (A)** Type I lobules demonstrated significantly different patterns of estrogen receptor (ER)α and progesterone receptor (PR) expression compared with other lobule types. Lobules were stained for ERα, PR, and Ki67, and the number of individual lobules in each category was quantified for each lobule type (n = 15 lobules/patient). Statistical differences were detected by chi-squared analysis. **(B)** Type I lobules demonstrated significantly reduced levels of EpCAM expression compared with other lobule types. Lobules were stained for EpCAM, and scored for cellular expression and intensity using Allred scoring criteria. The number of individual lobules in each category was quantified for each lobule type (n = 15 lobules/patient). Statistical differences detected by chi-squared analysis. Scale bar = 100 μm.

Given the difference in CK8/14 expression in Type I lobules compared to Type III lobules, we examined other markers associated with differentiation. Type I lobules have been reported to exhibit the highest percentage of estrogen receptor (ER) α and progesterone receptor (PR) positive [6],[28], as well as the highest proliferative index [7],[28]. Similar to the expression of CK8 and CK14, Type I lobules demonstrated significantly greater variability than Type II or Type III lobules for ERα and PR expression (Figure 5A). Consistent with previous reports [28], a higher proportion of the Type I lobules exhibited >20% cells expressing ERα and PR compared to the other lobule types (Figure 5A). We also assessed proliferation utilizing the marker Ki67. Although Ki67 expression was variable within the Type I lobule group, unlike ERα and PR expression, there were no significant differences among lobule types (Figure 5A). These results demonstrate significant heterogeneity within Type I lobules for multiple markers.

As Type I lobules demonstrated significant variations in ERα and PR expression as well as differences in CK8 and CK14 localization, we examined the expression of EpCAM. We quantified both the expression and intensity of EpCAM stain using the Allred scoring system (Figure 5B). Similar to the other markers examined, Type I lobules demonstrated variable staining with EpCAM, with some lobules enriched for an EpCAM^lo/neg^ population of epithelial cells (Figure 5B). Although a population of Type I lobules demonstrated EpCAM^lo/neg^ staining, these lobules still expressed PR, suggesting luminal rather than basal differentiation (Figure 5B). In total, these results suggest that epithelial cell within Type I lobules are less differentiated and enriched for progenitor populations. In addition, these findings suggest that Type III lobules are more mature, harboring more differentiated and lineage-committed epithelial cells.

### Complexity of the epithelial hierarchy trees defines lobule types

**Figure 6:**
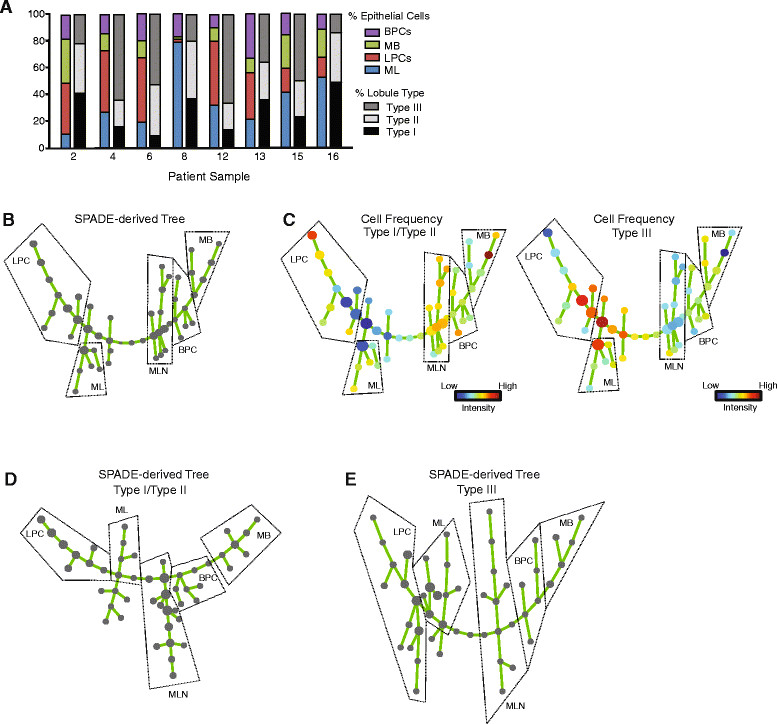
**Hierarchal trees reveal differences in the cellular hierarchy of Type I/Type II and Type III lobules. (A)** No correlations were found among specific cell populations, lobule type, or patient age. Percentage of epithelial cells in mature luminal (ML), luminal progenitor cell (LPC), mature basal (MB), and basal progenitor cell (BPC) populations assessed by flow cytometry analysis were compared to the percentage of each type of lobule found within the breast tissue for each patient sample (n = 8). **(B)** Spanning-tree progression analysis of density-normalized events (SPADE) was performed on flow cytometry data for protein markers EpCAM, CD24, and CD49f from eight patient samples with characterized lobule composition. Node size reflects the median number of cells in each population across the heterogeneous population. Dashed lines represent cell populations delineated by cell surface marker profiles. **(C)** The cell frequencies from breast samples with enrichment for Type I/Type II and Type III lobules were separated and visualized across the SPADE-derived tree. Nodes are colored by the median intensities of cell numbers in each node. Dashed lines represent cell populations delineated by cell surface marker profiles. **(D,** **E)** SPADE was performed on flow cytometry data for EpCAM, CD24, and CD49f for breast samples enriched for Type I/Type II **(D)** and Type III **(E)** lobules (n = 4 samples each). Dashed lines represent cell populations delineated by cell surface marker profiles. MLN, mammary lineage negative.

A major difficulty in delineating the epithelial hierarchy of the human breast has been the inability to equate progenitor populations defined *ex vivo* using cell surface immunophenotypes with their *in vivo* counterparts. As Type I lobules were enriched in less differentiated luminal and basal epithelial cells and Type III lobules were enriched in more mature lineage committed cells, we speculated that the proportion of ML, LPC, MB, and BPC might correlate with specific lobule types. Although we observed significant histological differences in CK8/14 expression patterning and EpCAM expression levels in Type I and Type III lobules, there was no single or combinatorial population of EpCAM/CD49f cells that correlated with any lobule distribution using standard flow cytometry analysis (Figure 6A, Additional file 1: Table S2).

A recent study has suggested that traditional data analyses for flow cytometry may obscure the underlying continuity of phenotypes that is inherent in cellular differentiation [29]. In order to further explore the relationship between lobule types and the epithelial hierarchy of the breast, we utilized a recently reported computational approach to objectively organize our flow cytometery data into a hierarchy of related phenotypes [19]. SPADE extracts a hierarchy from high-dimensional cytometry data in an unsupervised manner, which enables multiple cell types to be visualized in a branched tree structure in an unbiased manner [19]. Based on the local density of cell surface markers, SPADE mathematically reduces the number of cells represented in high-density regions, while maintaining the density of populations of rare cell types or cells in transition between the abundant cell types. Next, SPADE clusters each single cell with other cells based on the density of their cell surface markers and assigns each cell in the original data set to the generated clusters. The nodes generated by the SPADE analyses are of varying size, based on the number of cells represented within the node, and are colored by the median intensities of the cell surface markers represented by cells within that node.

SPADE was used to generate a hierarchal tree based on the eight reduction mammoplasty tissue samples that had been characterized by both flow cytometry and lobule composition (Figure 6B). Following SPADE, we examined the colored trees to manually identify the ML, LPC, MB or BPC populations represented by each part of the tree based on their known cell surface markers (Additional file 1: Figure S2A). We then determined the contribution of individual cell frequencies from less mature (Type I/Type II) and mature (Type III) lobules to the nodes comprising each defined population within the hierarchal tree (Figure 6C). Breasts with increased numbers of Type I/Type II lobules demonstrated a higher frequency of cells within the basal lineages as well as a group that was MLN (negative for EpCAM, CD24, CD49f), while those containing more Type III lobules were enriched in cells that were positive for luminal lineage markers (Figure 6C). These results demonstrate for the first time in the human breast a clear relationship between progenitor populations identified at the cellular level and structural features identified at the tissue level. Specifically, they suggest that differences in the cellular hierarchy exist between tissues predominantly consisting of mature or immature lobules.

To expand upon this idea, we further examined the hierarchal complexity by generating SPADE trees individually from Type I/Type II and Type III epithelial cells (Figure 6D, E; Additional file 1: Figure S2B, C). Following SPADE, we examined the colored trees to manually identify the ML, LPC, MB or BPC populations represented by each part of the tree (Additional file 1: Figure S2B, C). Interestingly, epithelial cells within the Type III lobules demonstrated a hierarchal tree of increased complexity compared to the tree formed by epithelial cells of Type I/Type II lobules (Figure 6D, E; Additional file 1: Figure S2B, C). Compared to Type I/Type II lobules, the epithelial hierarchy of the Type III lobules demonstrated multiple branches with smaller nodes, suggesting the presence of rare epithelial populations that cluster together (Figure 6E). These results suggest that with increasing glandular maturity, the epithelial hierarchy also becomes more complex.

## Discussion

The human breast undergoes extensive remodeling following birth through pregnancy and lactation, suggesting the presence of a cellular hierarchy to functionally expand the epithelium. Here, we demonstrate that alveolar and ductal structural progenitors exist and reside within luminal and basal lineages of the breast, respectively. Our findings strongly support a model of the breast epithelial hierarchy in which two types of phenotypically distinguishable progenitors contribute to specific structural elements of the mammary tree (ducts or alveoli) and that the combination of these progenitor cell populations is necessary for development and maintenance of mammary tissues. Luminal alveolar progenitors are more abundant in transplant assays and *in vitro* collagen assays than basal ductal progenitors, and SPADE demonstrated that the luminal lineages show increasing complexity with lobule maturity. These results suggest that different types of luminal alveolar progenitors may exist within the breast to functionally expand the lobules.

Stem/progenitor cells have been an important area of investigation for understanding the cellular origin of breast cancer (for review, [2]). Localization of stem/progenitor cells within human tissue has been challenging due to limitations on tissue availability, patient heterogeneity, and differences among techniques to measure stem/progenitor activity and cell surface markers used in studies. Multiple studies have examined stem/progenitor activity in populations defined by cell surface markers [11],[16],[20],[22],[30],[31], but have reported conflicting results about whether luminal or basal lineages were enriched for progenitor activity. Here, we show the relationships among the assays used to detect stem/progenitor activity, as well as their relationships with the cell surface markers most commonly used. Although there are important functional differences among the lineages, both the luminal and basal lineages retain distinct progenitor competency. Further, we show that Type I lobules, which are the least developed of the breast lobules and most closely associated with terminal ducts, are enriched in progenitor activity. Although we did not directly assess collecting ducts, our results are consistent with those found by Villadsen and colleagues who previously mapped progenitor activity to cells within main and terminal ducts, through use of collagenase digestion of breast epithelial organoids and microdissection [22].

Recent studies of the mouse epithelial hierarchy have incorporated numerous cell surface markers to characterize specific epithelial populations. However, determining the relationships among the cells identified by these markers has proven challenging. For the first time, using SPADE we identified specific differences between breasts enriched for Type I/Type II and Type III lobules that were obscured by traditional flow cytometry gating. Epithelial cells isolated from tissues enriched for Type I/Type II lobules demonstrated increases in basal lineages and mammary lineage negative cells, as well as different clusters of LPCs, compared with those from Type III lobules. Additionally, epithelial cells enriched from Type III lobules demonstrated increased clusters of mature luminal cells, which clustered into a more complex hierarchy when examined individually. Although recent studies have begun the integration of more cell surface markers to delineate the specific functions of different luminal epithelial cell types [30], the relationship between different luminal progenitor cells and lobule types were not examined. Identification and localization of these cell types in the context of lobule maturity may clarify functional studies of breast epithelial cells, where inherent breast heterogeneity often obscures consistent results.

Lineage tracing to physiologically identify and characterize progenitor cells in the mouse mammary gland has demonstrated that following birth, the expansion and maintenance of the luminal and basal lineages is ensured by the presence of lineage-restricted progenitor cells [9],[32]. Elegant studies have demonstrated that expression of both CK14 and CK8 were restricted to basal and luminal cells, respectively [9]. However, within human breast tissue, the presence of CK14^+^ epithelial cells within the luminal layer have been previously described [33]-[36], although the localization of CK14^+^ luminal cells was not evaluated with respect to lobule types. Here, we show that expression of CK8 and CK14 was variable in Type I lobules, with luminal expression of CK14 and basal expression of CK8 as well as co-expression of both cytokeratins in the luminal and basal layers. These results suggest that either human epithelial cell lineages are not as restricted as in the murine gland, or that CK8 and 14 expression is less specific to the luminal and basal lineages in the human as it is in the mouse. Although CK14 was present in luminal cells in Type I lobules, we did not identify any p63^+^ epithelial cells in this layer, suggesting that CK14 may not always be a precise marker for basal epithelial cells.

Russo and Russo have classified the degree of breast complexity into Type I, Type II, and Type III lobules [6]-[8]. The increasing lobular complexity during pregnancy in preparation for lactation suggests that the lobules become successively more mature in the progression from Type I to Type III. This implies that breast tissue from multiparous women contain an increased number of Type III lobules compared to the breasts of nulliparous women. Although mice undergo complete involution following pregnancy, resulting in parous mammary glands that are structurally very similar to nulliparous glands, the degree of involution in humans following pregnancy has not been well-characterized. Additionally, Type III lobules have been identified in very young nulliparous breast tissue [37],[38], which suggests that considerable heterogeneity exists among all patient samples, regardless of parity. Interestingly, in breast tissue from women undergoing elective reduction mammoplasty surgery, we observed surprising variability among lobules of the same patient for steroid receptor expression and proliferation, suggesting an underlying mechanism of regulation for lobule expansion. Even under the differentiating conditions of pregnancy, studies have noted that it is not unusual to see acini that are devoid of proliferative markers, while other acini demonstrate a dramatic proliferative response [37],[38]. Clearly, the mechanisms regulating the response of individual lobule types to hormonal stimuli require further investigation to determine why one lobule may remain quiescent, while others expand.

In this study, we did not observe any clear differences between Type I, II, and III lobules and age or parity, however, the number of patients examined in this study was small. Although we tried to obtain detailed reproductive information about the patients used in this study, we were not able to obtain information regarding the phase of the menstrual cycle or phase of oral contraceptive use at the time of surgery. Given the critical role that steroid hormones play in breast development [3],[6], changes circulating levels of progesterone and estrogen during the course of the menstrual cycle may influence the complexity of the epithelial populations present within the mammary gland as well as the structures of the lobules. A large study conducted with detailed questionnaires in addition to quantification of breast lobule types and epithelial cell surface markers would need to be conducted in order to fully address this issue.

Type I lobules have been characterized as being the least mature of the lobules types of the breast [6],[8]. Our results support this hypothesis, given the heterogeneity of expression of multiple markers within this lobule type. Interestingly, a recent study has demonstrated that breast tissue of aging women shows an increase in luminal CK14 expression [39], however, the localization of these cells was not directly examined within the lobules. Following menopause, the breast undergoes involution [40], which may increase the number of Type I lobules present within the tissue. Given that women older than 50 years account for the majority of new breast cancer diagnoses [40], examining the effect of aging on lobule composition may provide insight into the origin of breast cancer in this population. Studies examining the effects of carcinogens on tumorigenesis using rodent models have supported the idea that transformation of cells in Type I lobules give rise to the most common breast malignancies, whereas dysregulated growth in more mature lobules become benign breast lesions [41],[42]. However, a nested case-control study in the Nurses’ Health Studies demonstrated that women with predominantly Type I lobules within their breast had a decreased risk of breast cancer compared with those who had no Type I lobules or mixed lobule types [43]. Unraveling the normal growth regulation of specific lobules within the breast may provide insight into the dysregulated growth during cancer. As our data suggest underlying differences in the epithelial hierarchy between breasts with immature and mature lobules, studies examining the correlation between the lobule types present in the normal tissue matched with the specific breast tumor subtype could potentially clarify the differences between rodent and human models of breast cancer.

## Conclusions

In this study, we characterized progenitor cell activity in human breast tissue and identified the relationships among different assays to detect stem cells within the human epithelial hierarchy. Further, we elucidated the structural and anatomical location of undifferentiated progenitor cells within the breast lobules and identified specific differences in the cellular hierarchy that exist between tissues predominantly consisting of mature or immature lobules.

## Authors’ contributions

LMA participated in the study design and interpretation of the data, performed experiments, and participated in drafting the manuscript. PJK participated in study design and performed experiments. AS performed experiments and helped to draft the manuscript. KG performed experiments. SPN, RJB, HG and SEC participated in study design. CK participated in study design, interpretation of the data, and drafting the manuscript. All authors assisted in revision of the manuscript for intellectual content, read and approved the final manuscript, and agreed to be accountable for all aspects of the work in ensuring that questions related to the accuracy or integrity of any part of the work are appropriately investigated and resolved.

## Additional file

## Electronic supplementary material

Additional file 1: Figure S1.: Flow cytometry analysis for EpCAM, CD49f, and CD24. These data show the gating strategy, isotype controls and CD24 expression applied to Figures 1 and 6. **Figure S2.** Spanning-tree progression analysis of density-normalized events (SPADE) applied to epithelial cell flow cytometry data. These data show the EpCAM, CD24, and CD49f cell populations within the SPADE trees in Figure 6. **Table S1.** Origin of tissue collected from reduction mammoplasty surgeries. These data show the source and reproductive information on the patient tissue samples used in the study. **Table S2.** Percentage of mature luminal, luminal progenitor, mature basal, and basal progenitor cell populations. These data show individual EpCAM, CD24, and CD49f cell populations used in Figure 1. **Table S3.** Frequency of progenitor cells measured by *in vitro* assays of patient-derived mammary epithelial cells. These data show individual progenitor assay results for each patient sample examined summarized in Table 1. (PDF 221 KB)

Below are the links to the authors’ original submitted files for images.

Authors’ original file for figure 1

Authors’ original file for figure 2

Authors’ original file for figure 3

Authors’ original file for figure 4

Authors’ original file for figure 5

Authors’ original file for figure 6

Authors’ original file for figure 7

## Abbreviations

ANOVA: analysis of variance
BPC: basal progenitor cells
BSA: bovine serum albumin
CK: cytokeratin
DAB: 3,3-diaminobenzidine
DAPI: 4′,6-diamidino-2-phenylindole
DMEM: Dulbecco’s modified Eagle’s medium
DMSO: dimethyl sulfoxide
EGF: epidermal growth factor
ERα: estrogen receptor alpha
FACS: fluorescence-activated cell sorting
LPC: luminal progenitor cells
MB: mature basal
ME: myoepithelial
MEC: mammary epithelial cells
ML: mature luminal
MLN: mammary lineage negative
PBS: phosphate-buffered saline
PR: progesterone receptor
SMA: smooth muscle actin
SPADE: spanning-tree progression analysis of density-normalized events
TDLU: terminal ductal lobular units
