# Supplementary material for: Anatomical localization of progenitor cells in human breast tissue reveals enrichment of uncommitted cells within immature lobules
Source: Breast Cancer Res. 2014 Oct 15;16:453. doi: 10.1186/s13058-014-0453-3 (PMC4303132; doi:10.1186/s13058-014-0453-3)
Supplement: Supplementary file 1 — Additional file 1: Figure S1.: Flow cytometry analysis for EpCAM, CD49f, and CD24. These data show the gating strategy, isotype controls and CD24 expression applied to Figures 1 and 6. Figure S2. Spanning-tree progression analysis of density-normalized events (SPADE) applied to epithelial cell flow cytometry data. These data show the EpCAM, CD24, and CD49f cell populations within the SPADE trees in Figure 6. Table S1. Origin of tissue collected from reduction mammoplasty surgeries. These data show the source and reproductive information on the patient tissue samples used in the study. Table S2. Percentage of mature luminal, luminal progenitor, mature basal, and basal progenitor cell populations. These data show individual EpCAM, CD24, and CD49f cell populations used in Figure 1. Table S3. Frequency of progenitor cells measured by in vitro assays of patient-derived mammary epithelial cells. These data show individual progenitor assay results for each patient sample examined summarized in Table 1. (PDF 221 KB) [file 13058_2014_453_MOESM1_ESM.pdf]

Supplementary Table 1. Origin of tissue collected from reduction mammoplasty surgeries.

Patient	Age Range (yrs)	Year Collected	Use of Hormonal Contraceptives	Number of Pregnancies
1	>20	2007	Yes	0
2	>20	2009	Yes	Unknown
3	20-24	2010	Yes	0
4	20-24	2009	Yes	0
5	20-24	2009	No	Unknown
6	20-24	2009	Yes	0
7	25-29	2008	Unknown	Unknown
8	25-29	2009	Yes	0
9	25-29	2009	Unknown	Unknown
10	35-39	2007	Unknown	3
11	35-39	2008	Unknown	Unknown
12	35-39	2008	Mirena IUD	3
13	35-39	2009	Yes	3
14	40-44	2007	No, IVF treatment	0
15	40-44	2009	No, fertility treatment	2
16	50-54	2009	Unknown	Unknown
17	50-54	2007	Unknown	1
18	50-54	2007	Unknown	Unknown
19	55-59	2008	Unknown	Unknown

Supplementary Table 2. Percentage of Mature Luminal, Luminal Progenitor, Mature Basal, and Basal Progenitor Cell Populations.

Patient	Age Range (yrs)	EpCAM^{hi} CD49f^{-} (ML)	EpCAM^{hi} CD49f^{+} (LPCs)	EpCAM^{lo} CD49f^{+} (MB)	EpCAM^{-} CD49f^{+} (BPCs)
1	>20	57.0	13.1	13.6	16.3
2	>20	9.8	38.4	33.4	18.4
4	20-24	27.3	45.7	12.9	14.1
6	25-29	19.1	48.0	13.4	19.6
7	25-29	16.5	58.2	17.9	7.4
8	25-29	79.4	1.6	1.9	17.1
10	35-39	11.0	44.0	15.0	30.0
11	35-39	86.4	1.2	5.1	7.2
12	35-39	32.6	47.8	10.1	9.6
13	35-39	21.0	35.0	11.0	33.0
14	40-44	31.2	13.6	43.2	12.0
15	40-44	41.0	18.0	25.0	16.0
16	45-49	52.6	14.7	21.4	11.4
17	50-54	96.4	0.4	1.0	2.1
19	55-59	63.7	20.2	7.7	8.5

Supplementary Table 3. Frequency of progenitor cells measured by *in vitro* assays of patient derived mammary epithelial cells.

Patient	Age Range (yrs)	Freq Adherent Colonies	Enrichment of Colonies	Freq CK8/14 Colonies	Freq Mammo-spheres	Freq Floating Colonies	Freq 3D Colonies
1	>20	0.0074	Basal	0.00047	0.004	NT	NT
2	>20	0.0049	Basal	0.0017	0.0038	0.0023	0.0073
3	20-24	0.0066	Luminal	0.001	0.0039	0.0026	0.0006
4	20-24	0.00084	Basal	0.00028	0.004	0.0035	0.0063
5	20-24	0.015	Luminal	0.003	0.018	0.015	0.0043
6	25-29	0.0093	Basal	0.027	0.0069	0.0022	0.0024
7	25-29	0.0043	Luminal	0.00035	0.0028	0.0019	NT
8	25-29	0.042	Luminal	0.00045	0.0089	0.0082	0.0016
9	25-29	0.014	Basal	0.0016	0.011	0.0007	0.0016
10	35-39	0.0058	Luminal	0.00095	0.014	0.0081	NT
11	35-39	0.0094	Basal	0.0071	0.0051	0.0021	NT
12	35-39	0.0063	Luminal	0.00062	0.0078	0.0086	0.0026
13	35-39	0.003	Luminal	0.00008	0.0091	0.0073	0.0018
14	40-44	0.029	Basal	0.0037	0.0056	0.0033	NT
15	40-44	0.012	Basal	0.00043	0.0056	0.00035	0.0044
16	45-49	0.0072	Luminal	0.0003	0.0045	0.0025	0.00083
17	50-54	0.0018	Luminal	0.00028	0.0036	0.0044	0.001
18	50-54	0.0033	Luminal	0.0014	0.0067	0.0042	0.003
19	55-59	0.0041	Luminal	0.00048	NT	NT	NT

NT=not tested

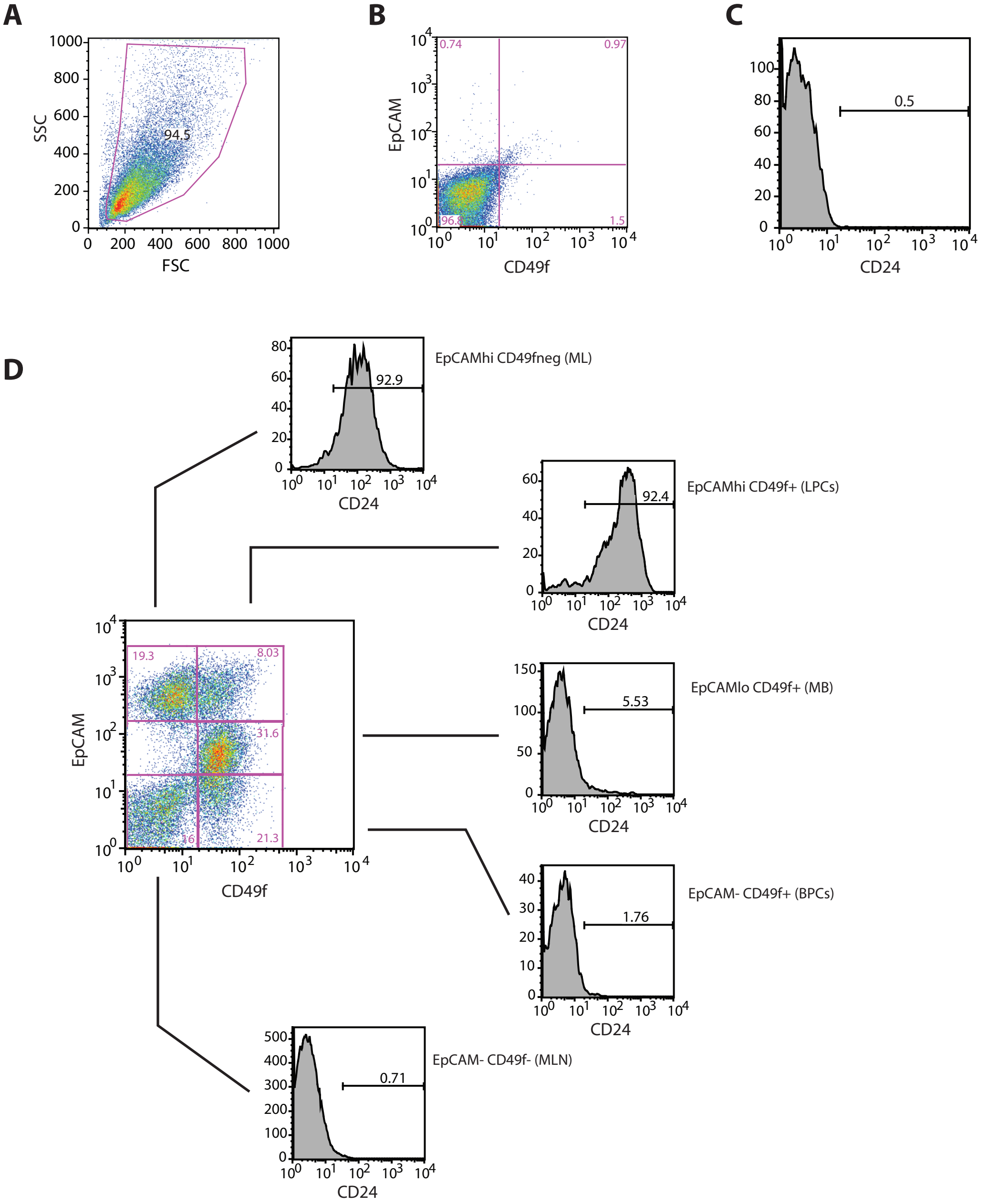
A
SSC
FSC
94.5
B
EpCAM
CD49f
0.74
0.97
96.8
1.5
C
CD24
0.5
D
EpCAMhi CD49fneg (ML)
92.9
EpCAMhi CD49f+ (LPCs)
92.4
EpCAMlo CD49f+ (MB)
5.53
EpCAM- CD49f+ (BPCs)
1.76
EpCAM- CD49f- (MLN)
0.71
19.3
8.03
31.6
16
21.3

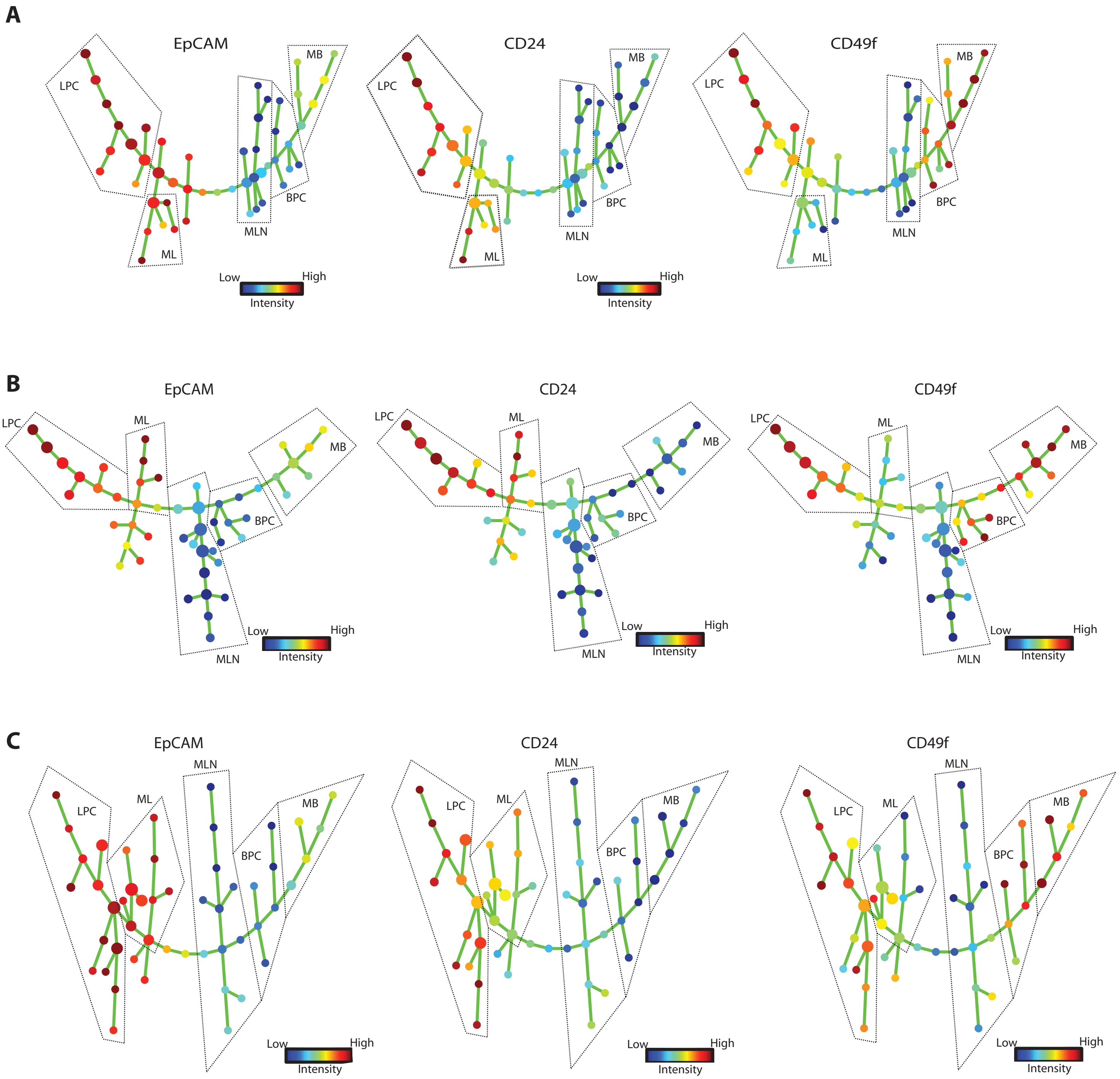
A
EpCAM
LPC
MB
ML
MLN
BPC
Low
High
Intensity
CD24
LPC
MB
ML
MLN
BPC
Low
High
Intensity
CD49f
LPC
MB
ML
MLN
BPC
B
EpCAM
LPC
ML
MB
BPC
MLN
Low
High
Intensity
CD24
LPC
ML
MB
BPC
MLN
Low
High
Intensity
CD49f
LPC
ML
MB
BPC
MLN
Low
High
Intensity
C
EpCAM
MLN
LPC
ML
MB
BPC
Low
High
Intensity
CD24
MLN
LPC
ML
MB
BPC
Low
High
Intensity
CD49f
MLN
LPC
ML
MB
BPC
Low
High
Intensity

Figure S1. Flow cytometry gating strategy for EpCAM, CD49f, and CD24. (A) Cell population gated to remove cellular debris and doublets. **(B)** Gates for $EpCAM^+$ and $CD49f^+$ cells based on isotype controls for each antibody. **(C)** Gate for $CD24^+$ cells based on isotype control for CD24 antibody. **(D)** Gates for each cellular population: Mature Luminal (ML) $EpCAM^{hi}CD49f^-$, Luminal Progenitor Cells (LPCs) $EpCAM^{hi}CD49f^+$, Mature Basal (MB) $EpCAM^{lo}CD49f^+$, Basal Progenitor Cells (BPC) $EpCAM^-CD49f^+$, Mammary Lineage Negative (MLN) $EpCAM^-CD49f^-$. CD24 expression denoted for each population.

Figure S2. SPADE applied to mammary epithelial cell flow cytometry data. (A) SPADE tree derived from the flow cytometry data of cell surface markers EpCAM, CD24, CD49f from patient samples with known lobule composition (n=8 patient samples). Trees are colored by the median intensity of each individual marker. Dashed lines denote cell populations based on cell surface markers: Mature Luminal (ML) $EpCAM^{hi}CD24^+CD49f^-$, Luminal Progenitor Cells (LPCs) $EpCAM^{hi}CD24^+CD49f^+$, Mature Basal (MB) $EpCAM^{lo}CD24^-CD49f^+$, Basal Progenitor Cells (BPC) $EpCAM^-CD24^-CD49f^+$, Mammary Lineage Negative (MLN) $EpCAM^-CD24^-CD49f^-$. **(B, C)** SPADE tree derived from the flow cytometry data of cell surface markers EpCAM, CD24, CD49f from patient samples enriched for Type I/Type II **(B)** and Type III **(C)** lobules types (n=4 patient samples/group). Trees are colored by the median intensity of each individual marker.
