# Supplementary figures and images for: Anatomical localization of progenitor cells in human breast tissue reveals enrichment of uncommitted cells within immature lobules
Source: Breast Cancer Res. 2014 Oct 15;16:453. doi: 10.1186/s13058-014-0453-3 (PMC4303132; doi:10.1186/s13058-014-0453-3)

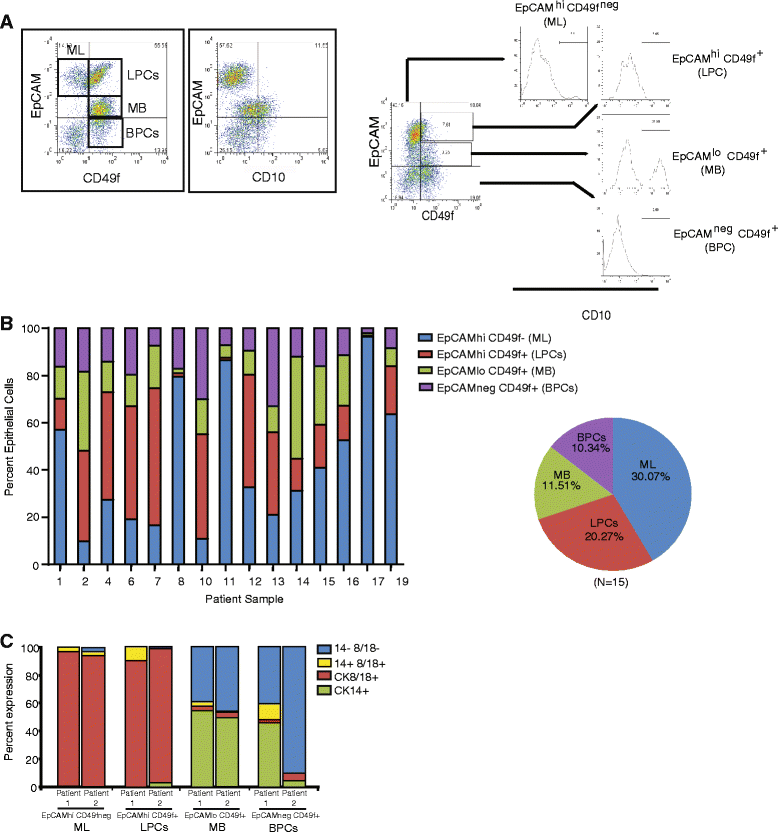

Supplement: Supplementary file 2 — Authors’ original file for figure 1 [file 13058_2014_453_MOESM2_ESM.gif]

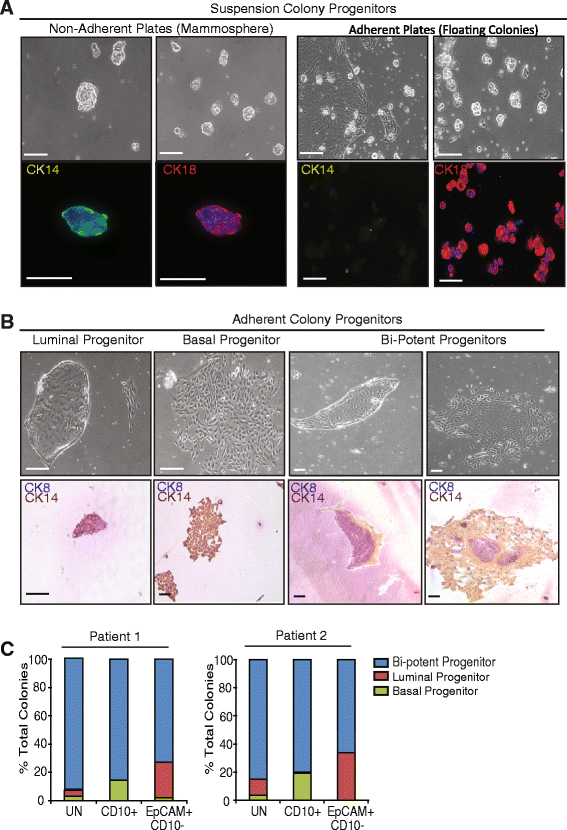

Supplement: Supplementary file 3 — Authors’ original file for figure 2 [file 13058_2014_453_MOESM3_ESM.gif]

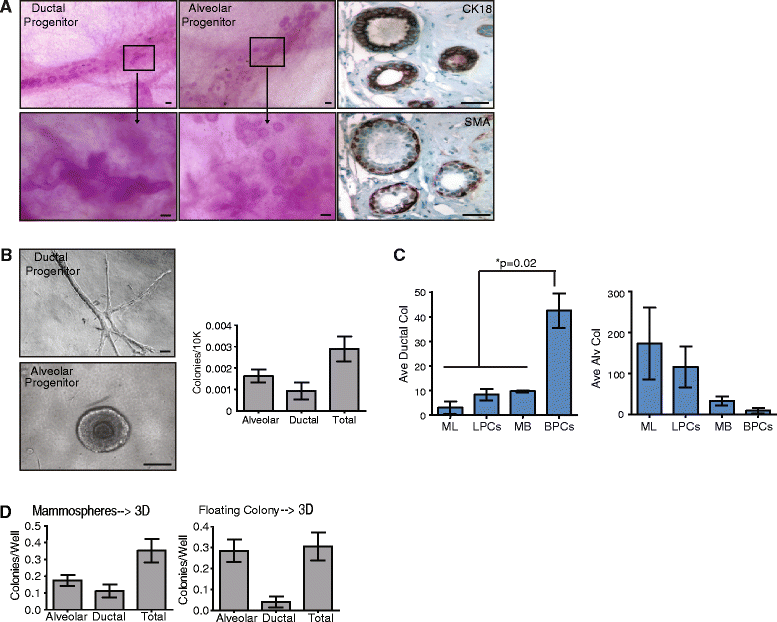

Supplement: Supplementary file 4 — Authors’ original file for figure 3 [file 13058_2014_453_MOESM4_ESM.gif]

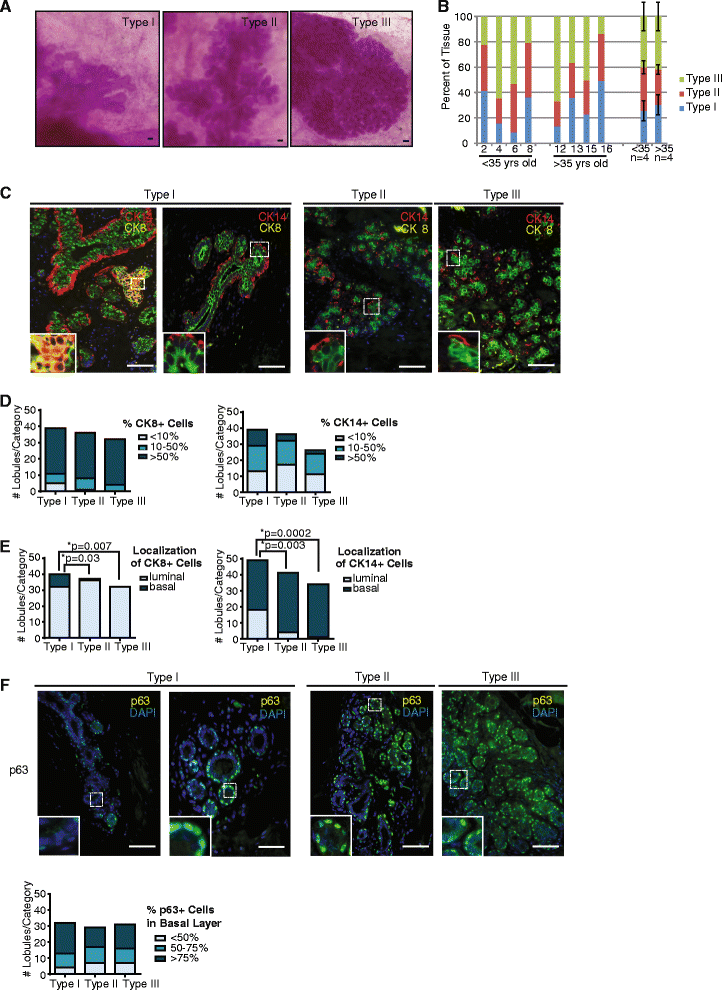

Supplement: Supplementary file 5 — Authors’ original file for figure 4 [file 13058_2014_453_MOESM5_ESM.gif]

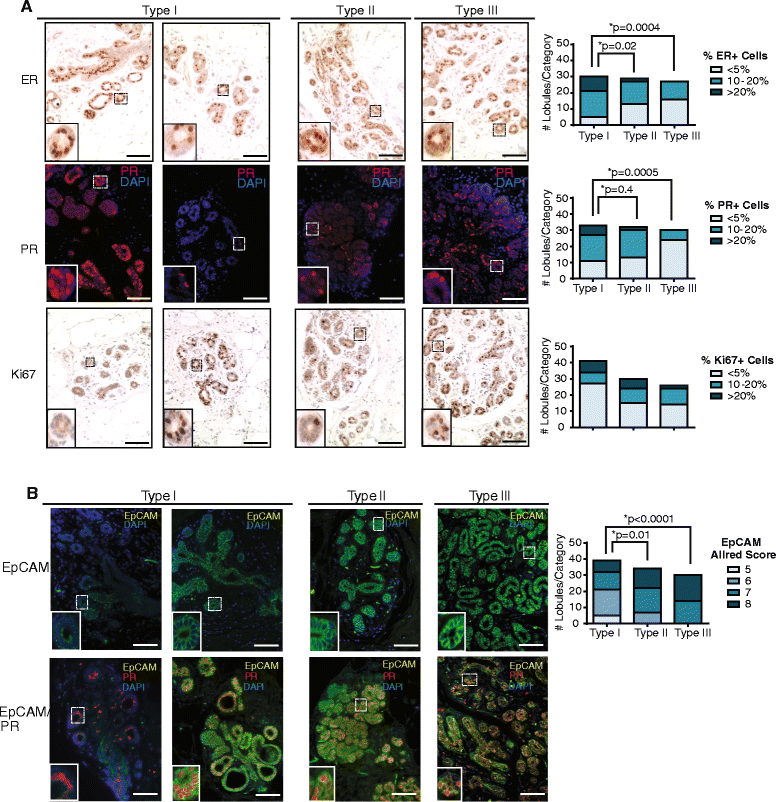

Supplement: Supplementary file 6 — Authors’ original file for figure 5 [file 13058_2014_453_MOESM6_ESM.gif]

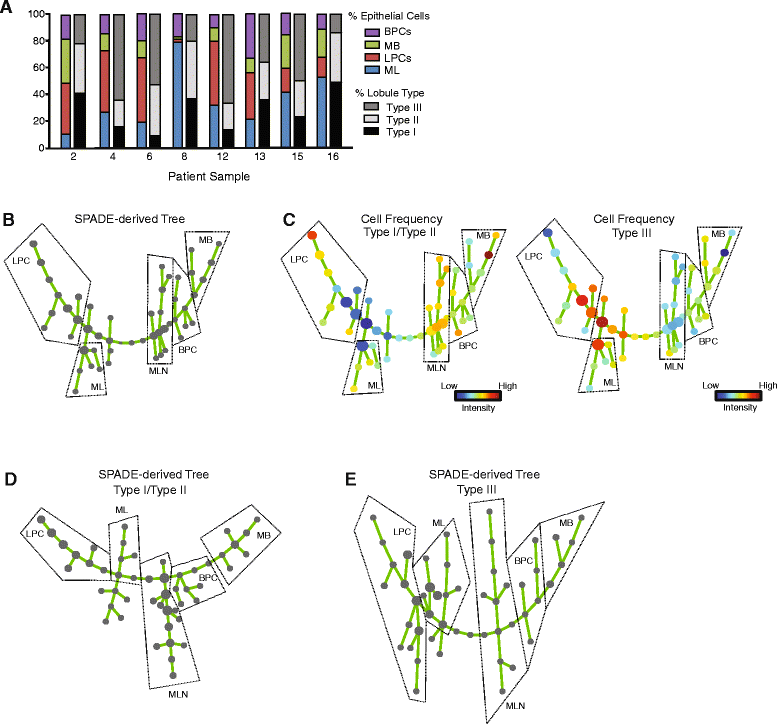

Supplement: Supplementary file 7 — Authors’ original file for figure 6 [file 13058_2014_453_MOESM7_ESM.gif]
