# Supplementary material for: Anatomical localization of progenitor cells in human breast tissue reveals enrichment of uncommitted cells within immature lobules
Source: Breast Cancer Res. 2014 Oct 15;16:453. doi: 10.1186/s13058-014-0453-3 (PMC4303132; doi:10.1186/s13058-014-0453-3)
Supplement: Supplementary file 8 — Authors’ original file for figure 7 [file 13058_2014_453_MOESM8_ESM.pdf]

Table 1. Summary of frequency of progenitor activity.

UNCULTURED CELLS			
Progenitor Type	Progenitor Lineage	Frequency	
Adherent progenitors (N=19)			
Total	Both	0.0098±0.0023	9.8/1000
Bi-potent	Both	0.002±0.0014	2/1000
Luminal Progenitors	Luminal	0.0036±0.004	3.6/1000
Basal Progenitors	Basal	0.0029±0.004	2.9/1000
Non-adherent progenitors			
Mammospheres (N=19)	Both	0.0067±0.00092	6.7/1000
Floating colonies (N=18)	Luminal	0.0048±0.00088	4.8/1000
Structural progenitors (N=13)			
Total		0.003±0.0006	3/1000
Luminal Alveolar Progenitor	Luminal	0.002±0.003	2/1000
Basal Ductal Progenitor	Basal	0.0009±0.0004	0.9/1000
Ave age: 34.5±12.6 yrs (mean±s.d.)			
